# Current and Emerging Treatment Options for Multidrug Resistant *Escherichia coli* Urosepsis: A Review

**DOI:** 10.3390/antibiotics11121821

**Published:** 2022-12-15

**Authors:** Mikaela M. Walker, Jason A. Roberts, Benjamin A. Rogers, Patrick N. A. Harris, Fekade B. Sime

**Affiliations:** 1 UQ Centre for Clinical Research, Faculty of Medicine, University of Queensland, Brisbane, QLD 4029, Australia; 2 Departments of Pharmacy and Intensive Care Medicine, Royal Brisbane and Women’s Hospital, Brisbane, QLD 4029, Australia; 3 Division of Anaesthesiology Critical Care Emergency and Pain Medicine, Nîmes University Hospital, University of Montpellier, 30029 Nîmes, France; 4 Monash Infectious Diseases, Monash Health, Melbourne, VIC 3168, Australia; 5 Centre for Inflammatory Diseases, Monash University, Melbourne, VIC 3168, Australia; 6 Pathology Queensland, Health Support Queensland, Herston, QLD 4006, Australia

**Keywords:** multidrug resistance, urosepsis, *Escherichia coli*, MRE

## Abstract

*Escherichia coli* is a versatile commensal and pathogenic member of the human microflora. As the primary causative pathogen in urosepsis, *E. coli* places an immense burden on healthcare systems worldwide. To further exacerbate the issue, multi drug resistance (MDR) has spread rapidly through *E. coli* populations, making infections more troublesome and costlier to treat. This paper aimed to review the literature concerning the development of MDR in uropathogenic *E. coli* (UPEC) and explore the existing evidence of current and emerging treatment strategies. While some MDR strains maybe treated with β-lactam-β-lactamase inhibitor combinations as well as cephalosporins, cephamycin, temocillin and fosfomycin, current treatment strategies for many MDR UPEC strains are reliant on carbapenems. Carbapenem overreliance may contribute to the alarming dissemination of carbapenem-resistance amongst some UPEC communities, which has ushered in a new age of difficult to treat infections. Alternative treatment options for carbapenem resistant UPEC may include novel β-lactam-β-lactamase or carbapenemase inhibitor combinations, cefiderocol, polymyxins, tigecycline, aminoglycosides or fosfomycin. For metallo-β-lactamase producing strains (e.g., NDM, IMP-4), combinations of cefazidime-avibacam with aztreonam have been used. Additionally, the emergence of new antimicrobials brings new hope to the treatment of such infections. However, continued research is required to successfully bring these into the clinic for the treatment of MDR *E. coli* urosepsis.

## 1. Introduction

Uropathogenic *Escherichia coli* (UPEC) is responsible for a number of diseases in humans including urinary tract infections (UTI), and urosepsis. Together, UPEC infections place an astounding burden on healthcare worldwide, causing 80–95% of community acquired UTI cases, and 27% of sepsis cases [1,2,3,4,5]. Antibiotics have been the mainstay of treatment in bacterial infections since their introduction in the early 20th century. However, the global dissemination of resistance has posed a challenge [6]. Multi-drug resistance is now one of the most critical challenges facing the world. UPEC infections are typically treated with β-lactam antibiotics, fluoroquinolones, aminoglycosides and trimethoprim-sulfamethoxazole [7,8,9]. However, the spread of third generation cephalosporin resistance mediated by Ambler class A and C β-lactamases, and carbapenem resistance from Ambler class A, B and D β-lactamases have rendered many antibiotics ineffective [10]. Still, some treatment options remain. While many class A and C β-lactamase producing strains are still susceptible to carbapenems, the increase in reliance on these last resort drugs has triggered an increase in the spread of carbapenem hydrolysing enzymes called carbapenemases [11,12,13,14]. Thus, finding carbapenem-sparing options is a priority. Older β-lactam-β-lactamase inhibitor (BLBLI) combinations such as amoxicillin/clavulanic acid may be effective against ESBL producing *E. coli* while newer BLBLIs such as the diazabicyclooctane (DBO) BLBLIs, may be active against AmpC producing *E. coli* [15,16]. New antibiotics are also in development, some of which demonstrated activity against KPC, NDM and OXA-48-like β-lactamases. This review aims to review the literature around multi drug resistance in UPEC and present a qualitative synthesis of existing evidence on current and future treatment options for MDR strains of UPEC.

## 2. Multi-Drug Resistance in UPEC

Multi-drug resistance is defined as non-susceptibility to at least one antimicrobial in three or more classes as tested with in vitro susceptibility testing [8]. While multi-drug resistance in *E. coli* was discovered in the late 20th century, the WHO has since categorised carbapenem resistant, extended spectrum β-lactamase (ESBL) producing *Enterobacterales* as priority one, with the fear being that antibiotic resistant *Enterobacterales* will be disproportionally prevalent by 2050 without new antibiotic discovery [17,18]. *E. coli* has since shown the ability to resist all classes of antibiotics used in treatment of UPEC infections with varying degree of resistance. β-lactams are the most common antibiotic family used to treat UPEC infections and work by inhibiting essential penicillin-binding proteins to disrupt cell wall synthesis [15,19]. β-lactam resistance is usually mediated by β-lactamases, enzymes able to hydrolyse antibiotics of this family and render them ineffective [15].

### 2.1. Extended Spectrum β-Lactamase

Some of the most clinically significant β-lactamases include variants of TEM, SHV and CTX-M enzymes [10]. These β-lactamases are classified into one of four Ambler classes, with TEM, SHV and CTX-M categorised as Class A β-lactamases [20]. Both TEM-1 and SHV-1 are narrow spectrum β-lactamases, resulting in the hydrolysis of penicillins and cephalosporins such as cefamandole and cefoperazone. The wide use of extended-spectrum cephalosporins led to the emergence of mutants derived from TEM-1 and SHV-1 [10]. These new variants possess an extended spectrum of activity, mediating resistance to second-, third-, and fourth-generation cephalosporins, hence termed ESBLs [21]. CTX-M is the most widespread ESBL, first discovered and most prevalent in *E. coli* (though it is believed that CTX-M originated in *Kluyvera* spp.). The rapid global dissemination of CTX-M has since been referred to as the CTX-M pandemic [22,23].

ESBLs are defined by their ability to hydrolyse three β-lactam classes; penicillins, cephalosporins (including broad spectrum agents such as ceftriaxone and ceftazidime) and monobactams, but not cephamycins or carbapenems [24,25]. ESBLs often exhibit resistance to multiple other antibiotics such as aminoglycosides and fluoroquinolones via transmission of co-resistance located on the same mobile genetic elements, making these infections even more difficult to treat [26].

### 2.2. Plasmid-Mediated AmpC-β-Lactamase

AmpC-β-lactamases are classified as Ambler Class C [20]. AmpC-β-lactamases are usually chromosomally encoded, though *E. coli* only produces low and usually ineffectual levels, with the exception of some mutant strains [27]. However, AmpC-β-lactamases may also be transferred via plasmids [28,29]. AmpC β-lactamases provide resistance to all β-lactams with the exception of cefepime, cefpirome, and the carbapenems [25,30,31]. AmpC-β-lactamases are not as wide-spread as ESBLs with numerous studies reporting less than 7% of isolated *E. coli* containing AmpC-β-lactamase [32,33,34,35,36,37,38,39,40]. Colonisation of AmpC-β-lactamase producing *E. coli* strains is strongly associated with healthcare contact [35]. 

### 2.3. Carbapenemase 

Carbapenemases span three of the four Ambler classes.

Class A carbapenemases can have a chromosomal location but are often plasmid-mediated [11]. *K. pneumoniae* carbapenemase (KPC), which is mainly seen in *Klebsiella*, is also the most predominant type of class A carbapenemases in *E. coli* [14]. KPCs are broad-spectrum, acting against almost all β-lactams.

Class B carbapenemases are broad-spectrum β-lactamases able to hydrolyse all β-lactam antibiotics that are currently clinically available with the exception of monobactams [9,41]. The most common metallo-β-lactamases (MBL) reported in *E. coli* are variants of New Delhi metallo-β-lactamase (NDM), which was first observed in *E. coli* in 2009 [14].

Class D carbapenemases are types of oxacillin-hydrolysing enzymes [42]. Of the >200 enzymes, only a few are active against carbapenems with OXA-48 and OXA-48-like such as OXA-181, OXA-232 and OXA-484, being the most common in *E. coli* [43,44]. These carbapenemases have weak activity by themselves, and often spare cephalosporins, however co-occurrence with other resistance mechanisms including ESBLs can result in high-level third generation cephalosporin and carbapenem resistance [45].

### 2.4. Resistance to Non-β-Lactam Antibiotics

Though β-lactam antibiotics are the drug of choice for treating UPEC infections, other classes of antibiotics are also effective against *E. coli* [46]. These include fluoroquinolones, aminoglycosides, and fosfomycin as well as sulfonamides and trimethoprim [9]. Various resistance mechanisms also exist to impact the efficacy of these drugs and are often found in ESBL producing strains [26,47]. 

#### 2.4.1. Fluoroquinolones 

**Figure 1:**
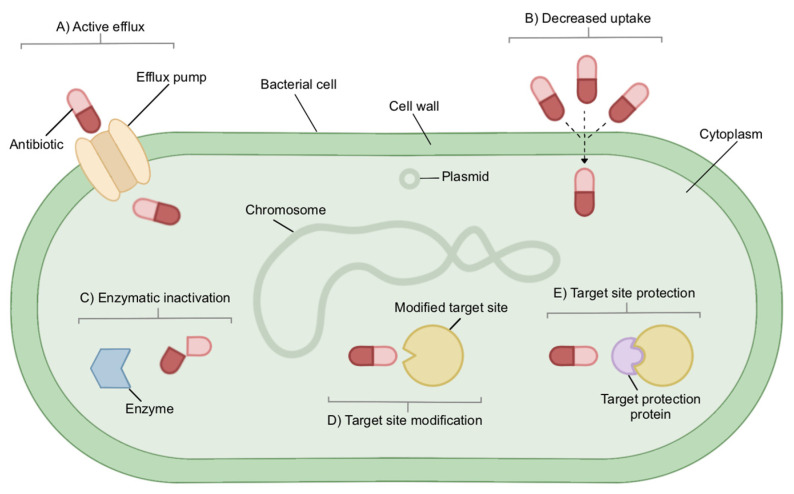
Possible mechanisms of resistance include (**A**) activation of efflux pumps, which remove drugs such as β-lactams from the bacterial cell (**B**) decreased drug uptake, preventing drugs such as fosfomycin from entering the cell (**C**) enzymes such as β-lactamase, which inactivate antibiotics, (**D**) modification of target site disallowing binding of drugs such as sulfonamides and trimethoprim and (**E**) protection of target site inhibiting binding of drugs such as fluroquinolones.

Fluoroquinolones are active against a wide range of bacteria [46,48]. They work by interfering with DNA supercoiling [46]. Resistance is commonly due to mutations in drug targets, though upregulated efflux pumps, protection of target structures and reduced permeability of the outer membrane are all thought to play a role in resistance (Figure 1) [46]. A 2016 study from the US found that 28.2% of tested strains were resistant to fluoroquinolones, while a 2019 study in Iran found 45.2% of their isolates were fluoroquinolone resistant [49,50]. Though fluoroquinolones are not a first-line treatment in UTI cases, reports show their prescription in nearly 50% of uncomplicated UTI cases [51]. Reports also show a direct correlation between floroquinolone use and community resistance rates [52].

#### 2.4.2. Aminoglycosides 

Aminoglycosides are often used in combination with β-lactams in UPEC infections. They work by irreversibly binding to the 30S subunit of the 16S rRNA, as well as the 50S subunit of the 70S bacterial ribosome [46,53]. In some bacteria, resistance may occur by mutation in the 16S RNA, and/or S5 and S12 ribosomal protein [53]. The likelihood of such a mutation occurring in *E. coli* is low, due to the numerous copies of these operons that *E. coli* possesses [54]. For this reason, resistance to aminoglycosides in UPEC is plasmid mediated [46]. Resistance to aminoglycosides can also be caused by enzymatic inactivation or protection of ribosomal methylases [55]. 

#### 2.4.3. Fosfomycin 

Fosfomycin is a broad spectrum antibiotic effective on many Gram-positive and Gram-negative bacteria. There are three forms of the antibiotic, two of which are oral formulations—fosfomycin tromethamine and fosfomycin calcium—and one IV formulation, fosfomycin disodium [56]. While oral fosfomycin is used worldwide, IV fosfomycin has yet to obtain FDA approval in the United States, despite being used in other countries for treatment of severe infections due to MDR Gram negative pathogens [16,57,58]. Fosfomycin works by inhibiting peptidoglycan synthesis and is often used to treat cystitis [8]. There are two major resistance mechanisms against fosfomycin, including mutations encoding for proteins involved in the uptake system, and the acquisition of plasmids encoding for fosfomycin-modifying enzymes [46].

#### 2.4.4. Sulfonamides and Trimethoprim 

Sulfonamides and trimethoprim are bacteriostatic antibiotics that interfere with bacterial folic acid synthesis, and are synergistic when given in combination [46]. Resistance is a result of either a mutation in the gene encoding for target enzymes, or the acquisition of genes that are insensitive to either sulfonamides or trimethoprim [46].

## 3. Current Treatment Options for MDR UPEC

Initial selection of antimicrobials is often determined by the suspected pathogen and its susceptibilities [59]. However, studies suggest that empiric treatment for patients with ESBL-producing infections have low appropriateness rates ranging from 37–50% [60]. Inappropriate therapy has been associated with increased morbidity and mortality, lengthening hospital stay and contributing to the development of AMR [51,61]. Recommended empiric treatment varies by location and should be guided by local susceptibility trends.

### 3.1. ESBL and AmpC Producing UPEC

#### 3.1.1. Carbapenems

As both ESBL and AmpC producing UPEC are typically resistant to most cephalosporins, carbapenems have traditionally been recommended to treat such infections [16]. Most literature seems to focus on meropenem and imipenem, with a fewer number studying ertapenem, as comparators for non-carbapenem drugs intended for use against ESBL and AmpC producing UPEC. As the dependence on carbapenems may be, in part, to blame for the increasing rates of carbapenem resistance seen in recent years, where possible, carbapenem sparing options should be favoured.

#### 3.1.2. β-Lactam-β-Lactamase-Inhibitor Combinations

β-lactam-β-lactamase-inhibitors (BLBLI), are one therapy of treating ESBL producing *Enterobacterales* [16]. BLBLIs effective against Class A enzymes include, amoxicillin-clavulanate, and piperacillin-tazobactam among others [15,16]. However, whether BLBLIs truly present a comparable treatment to carbapenems is still unclear. In a retrospective observational study of ESBL producing *E. coli* bloodstream infections, Rodríguez-Baño et al. observed a 9.3% mortality rate in patients receiving a definitive treatment of either amoxicillin-clavulanate and piperacillin-tazobactam versus 16.7% receiving carbapenems (*p* > 0.2) [62]. However, in this study, the treatment groups were unequal, with patients treated with carbapenems having higher illness severity. Conversely, in a noninferiority randomised clinical trial (MERINO), Harris et al. found that the 30-day mortality rate for patients with bloodstream infection caused by ESBL or AmpC-producing *E. coli* or *K. pneumoniae* who were treated with piperacillin-tazobactam was 12.3% versus 3.7% (*p*  =  0.90) in patients treated with meropenem [63]. While multiple studies and systematic reviews have reported that BLBLIs present a promising alternative to carbapenem use [62,64,65,66,67,68,69,70], many others have conversely shown carbapenems remain the superior treatment [63,71,72]. Additional studies are required to confirm BLBLI noninferiority for different infection syndromes, leaving carbapenems as the drug of choice to combat ESBL producing bacteria. One such additional study is a phase 4 clinical trial aiming to replicate the MERINO trial in lower risk patients. The study is currently recruiting and estimated to be completed in April 2024 [73].

Traditionally, AmpC-producing *Enterobacterales* possess enzymes that are not well inhibited by BLBLIs, however, second generation BLBLIs such as ceftolozane-tazobactam and ceftazidime-avibactam have been designed to combat AmpC-producing *Enterobacterales* [16,24]. As is the case with BLBLIs for ESBL producers, it remains unknown whether these new BLBLIs are a comparable treatment to carbapenems. A 2019 randomised clinical trial comparing ceftazidime-avibactam against the best available therapy (BAT) showed that clinical cure rates between the two were similar (80.0% ceftazidime-avibactam, 89.5% BAT) [74]. A 2020 systematic review of six randomised controlled trials showed that pooled data of ceftazidime-avibactam, cefiderocol, plazomicn and eravacycline was superior to carbapenems in composite cure, a combination of clinical success and microbiological eradication, though not statistically significantly so [75]. However, a 2021 systematic review and meta-analysis of six randomised controlled trials showed that ceftolozane-tazobactam and ceftazidime-avibactam had a lower clinical and microbiological success rate when compared to carbapenems [76]. 

#### 3.1.3. Cephalosporins

While both ESBL and AmpC producing *Enterobacterales* are resistant to most cephalosporins, exceptions do exist, though the literature is mixed. TEM and SHV producing ESBLs have shown susceptibility to cefotaxime in vitro though it is not used in practice, while CTX-M producers can be susceptible to ceftazidime and cefepime [16]. 

In a randomised controlled trial, Seo et al. found that the clinical and microbiological response to cefepime was only 33.3% compared to that of piperacillin-tazobactam (93%) and ertapenem (97%) (*p* < 0.001) in the treatment of ESBL-producing UPEC causing UTI [77]. Similarly, in a systematic review of thirty-five studies, Son et al. found little difference in mortality with empirical treatment of cephalosporins and carbapenems, with a risk ratio (RR) of 0.72 (0.11–1.17), although, the mortality rate was lower in the carbapenem group versus the cephalosporins, RR = 0.56 (0.42–0.74), with regard to definitive treatment [64]. However, in a single centre retrospective study in patients with UTI caused by ESBL producing *Enterobacterales*, Kim et al. found that cefepime was comparable to carbapenems. It should be noted that in this study, the sample sizes were very small, and drastically fewer patients received cefepime (n = 17) compared to carbapenems (n = 89) [78]. 

Additionally, chromosomally encoded AmpC producers may be susceptible to cefepime, though there is little clinical data supporting susceptibility in plasmid-mediated AmpC producers [16]. Overall, current literature suggests that cephalosporin monotherapy may be suboptimal to carbapenem when treating ESBL and AmpC-producing UPEC, and are often recommended to be avoided.

#### 3.1.4. Cephamycins

Cephamycins are second-generation cephalosporins active against ESBL producers, though they are not active against AmpC-producers [79]. Cephamycins include cefoxitin, cefotetan, cefmetazole, moxalactam and flomoxef. In a retrospective observational study, Araki et al. found that there was no significant difference (*p* = 0.72) in clinical cure rate between cefmetazole (86.1%) and meropenem (89.5%) when treating ESBL producing *E. coli* and *K. pneumoniae* [80]. Similarly, another retrospective observational study described comparable efficacy of cefmetazole and carbapenems in the treatment of UTI caused by ESBL-producing *E. coli*, though the authors did conclude that carbapenems were the preferred drug of choice for more severe cases [81]. However, in cefotaxime-resistant bacteria, use of cephamycins were associated with a longer hospital stay (10.2 ± 8.5) when compared to a carbapenem (14.6 ± 9.4 days) (*p* < 0.01), though the 28 day mortality rates were not statistically significant (20.7% vs. 13.8%, respectively, *p* = 0.28) [82]. Conversely, Lee et al. found that flomoxef had a significantly higher mortality rate than carbapenems (27.3% vs. 10.5%, *p* < 0.01) in the treatment of sepsis caused by ESBL-producing UPEC [83]. Therefore, although cephamycins present a possible carbapenem sparing option for non-severe infections, additional clinical data are still needed.

#### 3.1.5. Temocillin

Temocillin is a penicillin antibiotic first developed in the 1980s, before being relaunched in Germany in 2019 [84]. Temocillin is marketed as a parenteral therapy of bloodstream infections and complicated urinary tract infections, stable against both ESBL and AmpC-producers [84]. In a retrospective observational study of adults with a UTI caused by ESBL-producing UPEC, Delroy et al. found that 94% of the 72 patients who were treated with temocillin reached clinical cure compared to 99% of the 72 patients treated with a carbapenem (*p* = 0.206) [85]. However, of these patients only six were given temocillin as a first line therapy, others were first given a carbapenem, before being switched to temocillin [85]. This suggests that temocillin may be a potential treatment for ESBL and AmpC producing UPEC causing UTI. A phase 3 clinical trial is currently recruiting to compare the efficacy of temocillin versus meropenem against third-generation cephalosporin resistant *Enterobacterales* [86].

#### 3.1.6. Intravenous Fosfomycin

In a multicentre, double-blind, randomized, noninferiority trial Kaye et al. compared intravenous fosfomycin to piperacillin-tazobactam to treat complicated UTIs and found fosfomycin had a 64.7% overall success rate compared to 54.5% of piperacillin-tazobactam [87,88]. However, in a multicentre clinical trial of patients with urosepsis caused by UPEC, Sojo-Dorado et al. found only 68.6% of fosfomycin patients reached clinical and microbiological cure versus 78.1% patients treated with meropenem or ceftriaxone (*p* = 0.10) [89]. This suggests fosfomycin monotherapy is not as efficacious as other comparators. 

Recent studies have shown that fosfomycin has great success when used in combination with other antibiotics. In a prospective, multicentre, non-interventional study in 209 patients with ESBL-producing bacteria, including *E. coli*, Putensen et al. found that 81.3% of patients had an overall clinical success when treated with fosfomycin in combination, predominantly with a carbapenem, 3rd- or 4th- generation cephalosporin, BLBLI, and/or a quinolone [90]. Of these 209 patients, 51 patients had at least one MDR pathogen, and an even higher rate of success (84.8%) was observed in these patients [90]. Additionally, a combination of fosfomycin with colistin has been shown to be synergistic against MDR UPEC in vitro [91]. In combination, fosfomycin presents a promising alternative to other treatments.

### 3.2. Carbapenemase Producing UPEC

Treatment options for carbapenemase producing UPEC that are supported by clinical evidence are far more limited (Table 1). As such, an individualised approach is often necessary.

#### 3.2.1. Carbapenems

Though some studies show select carbapenems may act as a sufficient treatment, more data is available on the efficacy of carbapenem when used with BLIs [16]. The carbapenem, meropenem combined with the BLI vaborbactam, has been studied for the treatment of cUTI and associated bacteraemia [96]. Vaborbactam is a first-in-class boronic acid β-lactamase inhibitor that inhibits KPC enzymes, which was recently introduced to clinical practice [97]. In a phase 3, multicentre, multinational, randomised clinical trial (TANGO I) Kaye et al. found overall success occurred in 189 of 192 (98.4%) patients treated with meropenem-vaborbactam versus 171 of 182 (94.0%) patients treated with piperacillin-tazobactam (*p*  <  0.001) [98]. *E. coli* was the causative agent in 65.1% of patients treated with meropenem-vaborbactam and 64.3% of patients treated with piperacillin-tazobactam, however whether these pathogens were confirmed as CRE was not noted in the study [98]. Additionally, another phase 3, multinational, randomised controlled trial (TANGO II) confirmed that meropenem–vaborbactam monotherapy resulted in increased clinical cure, decreased mortality, and reduced nephrotoxicity compared to the BAT, which was either a monotherapy or combination therapy of polymyxins, carbapenems, aminoglycosides, or tigecycline; or monotherapy with ceftazidime-avibactam [92].

Imipenem/cilastatin/relebactam has been studied for the treatment of cUTI [99]. Literature on imipenem/cilastatin/relebactam against carbapenem resistant *E. coli* is scarce, however, a phase 3 clinical trial in patients with hospital-acquired pneumonia/ventilator-associated pneumonia, complicated urinary tract infections, or complicated intraabdominal infections which were caused by imipenem non-susceptible Gram negatives other than *E. coli*, found that there was a 28 day favourable clinical response in 71% of imipenem/cilastatin/relebactam patients versus 40% in colistin-imipenem patients [100]. This study also showed imipenem/cilastatin/relebactam had fewer serious adverse effects than the comparators, resulting in a promising treatment for carbapenem resistant *Enterobacterales*.

#### 3.2.2. Non-Carbapenem β-Lactams

There are few non-carbapenem β-lactams that present potential treatment options for carbapenemase producing *E. coli*, though clinical studies are scarce. Cefiderocol is a novel cephalosporin with activity against carbapenem resistant UPEC across all Ambler classes, including ESBL-, AmpC-, KPC- and NDM-1-producing strains [101]. While most clinical studies of cefiderocol have been conducted against *K. pneumoniae*, *Acinetobacter baumannii*, and *Pseudomonas aeruginosa*, in vitro studies against *E. coli* are promising. In a multicentre phase 3 study of patients with nosocomial pneumonia, bloodstream infections, or complicated UTI caused by carbapenem-resistant Gram negatives, Bassetti et al. found microbiological eradication was achieved by 53% of cefiderocol patients with UTI and 20% of patients in the BAT group with UTI [93]. However, *E. coli* accounted for very few of the patients in this study [93]. Additionally, a multicentre phase 2 non-inferiority trial in patients with UTI caused by a wide variety of carbapenem-resistant pathogens, including *E. coli*, found cure in 73% of patients treated with cefiderocol versus 55% of patients treated with imipenem-cilastatin (*p* = 0.0004) [102]. Cefiderocol is a promising carbapenem sparing option.

While there have been no randomised controlled trials as to the efficacy of temocillin to treat carbapenemase producing *E. coli*, it presents an interesting carbapenem sparing option. Though temocillin is hydrolysed by both OXA-48 and Ambler class B β-lactamases, studies have shown some retained efficacy against KPC producing isolates [103]. However, in addition to studying the efficacy of temocillin against ESBL and AmpC producing *Enterobacterales*, Kuch et al. also looked at the effects of temocillin on KPC producing isolates, and found 42.5% were susceptible to temocillin using a UTI breakpoint, however 100% were resistant using a systemic infection break point [104].

Another promising β-lactam antibiotic is aztreonam. Emeraud et al. found that 86% of MBL-producing *Enterobacterales* isolates were susceptible to combinations of aztreonam-ceftazidime-avibactam in vitro, while 50% were susceptible to aztreonam-amoxicillin-clavulanate, and 20% were susceptible to aztreonam-ceftolozane-tazobactam [105]. Additionally, a systematic review found that aztreonam-avibactam had an 80% efficacy against MBL-producing *Enterobacterales* in vitro [106]. Numerous studies conducted in MBL-producing *K. pneumoniae* support these findings, resulting in a promising alternative treatment [105,106,107,108,109,110,111,112,113].

#### 3.2.3. Polymyxins

Polymyxin B and polymyxin E (colistin) are last resort drugs, and one of the most common choices to treat carbapenem resistant *Enterobacterales* [16]. Polymyxin B and colistin may both be administered parenterally, however colistin is administered as colistin methanesulfonate (CMS), an inactive pro-drug, resulting in necessary in vivo conversion, which causes lower and slower plasma concentration compared to polymyxin B [114]. While polymyxin B is often the preferred choice to treat most infections, colistin is often the preferred polymyxin to treat UTI as it is believed conversion of CMS to colistin occurs in the urine, resulting in higher urinary levels of CMS [114,115].

Numerous studies have demonstrated the emergence of other drugs that appear to be more effective than polymyxins against carbapenem-resistant bacteria. For example, the previously mentioned phase 3 trial comparing imipenem/cilastatin/relebactam to colistin-imipenem, saw imipenem/cilastatin/relebactam was significantly superior to colistin-imipenem [100]. Additionally, in a prospective, multicentre, observational study, the efficacy and safety of colistin was compared to a new BLBLI combination ceftazidime-avibactam (discussed below) [116]. Of the 38 patients suffering from carbapenem resistant *Enterobacterales* bloodstream, urinary tract and respiratory tract infections included in the trial, patients treated with colistin had a 32% mortality rate versus just 9% in ceftazidime-avibactam (*p* = 0.001) [116].

Comparative monotherapy between colistin and other first-line drugs is controversial due to the severe nature of many illnesses in which colistin is used and other first-line drugs are not [16]. Colistin continues to be an option for adult UPEC infections while alternatives are in development.

#### 3.2.4. Tigecycline

Tigecycline is unaffected by ESBLs and AmpC β-lactamases and is often used as a last-resort drug as it is effective against carbapenemase producing organisms in vitro [117,118,119,120,121,122]. However, in practice, the pharmacokinetic properties of tigecycline present difficulties for treating UTI, due to the limited urinary excretion of the unchanged drug [123,124,125] Thus, studies investigating the efficacy of tigecycline in treating *E. coli* urosepsis are extremely limited. In a systematic review of 14 cases of MDR UTI, Brust et al. found positive clinical outcome in 78% of cases, with concomitant antibiotics including carbapenems colistin, or aminoglycosides [126]. These cases included UTIs caused by ESBL-producing *E. coli* and *K. pneumoniae*, KPC- producing *K. pneumoniae*, MDR *A. baumannii* and *E. aerogenes*, as well as Vancomycin-resistant Enterococci [126]. Sader et al. collected 12,942 *E. coli* isolates, of which 27 were meropenem-nonsusceptible, and found that 100% of their meropenem-nonsusceptible *E. coli* strains were susceptible to tigecycline [127].

Despite this, due to its poor renal clearance, tigecycline is not recommended for the treatment of UTI. Additionally, tigecycline has a high mortality rate, and due to this, the FDA has issued a boxed warning instructing clinicians to reserve tigecycline for situations where other treatments are not available [128,129,130,131,132].

#### 3.2.5. Aminoglycosides

Aminoglycosides are often used in combination with other antibiotics to treat carbapenem resistant strains. Increasing resistance to aminoglycosides has been observed in NDM and KPC producers. However, a relatively new aminoglycoside, plazomicin, has shown interesting results in trials. Plazomicin is an IV semisynthetic aminoglycoside approved for the treatment of cUTI [133]. A multicentre, multinational, randomized, double-blind, phase 3 trial comparing plazomicin to meropenem in MDR (including carbapenem resistant) pathogens of which *E. coli* predominated, showed that plazomicin was noninferior to meropenem [134]. At the test-of-cure visit, 81.7% of patients in the plazomicin group were found to have composite cure (combined microbiological eradication and clinical cure), versus 70.1% of the meropenem group. Though this trial did not focus solely on carbapenemase-producing *E. coli*, and some strains included were imipenem or doripenem resistant, but meropenem susceptible. Plazomicin presents a possible future option for the treatment of carbapenem-resistant *E. coli*, however more evidence from clinical studies is needed to confirm any clinical benefit [134].

#### 3.2.6. Fosfomycin

In addition to being frequently used in treatments of ESBL and AmpC-producing *Enterobacterales*, fosfomycin is frequently used as a part of combination therapy to treat carbapenem resistant strains, though literature on the efficacy of fosfomycin monotherapy are scarce [16]. A multicentre, observational, prospective case-series study of IV fosfomycin against extensively drug resistant ventilator assisted pneumonia, catheter-related bloodstream infections, intra-abdominal infections, and primary bacteraemia found that bacterial eradication occurred in 56.3% of cases [135]. Extensively drug resistant pathogens are susceptible to only one or two antimicrobial classes, and resistant to all others [8]. Though this study was not conducted in *E. coli*, it is one of very few that investigate fosfomycin monotherapy.

#### 3.2.7. Ceftazidime-Avibactam

The BLBLI combination of ceftazidime-avibactam has shown promising results in vitro, with activity against most KPC and OXA-48 producing *Enterobacterales*. In a single-centre retrospective observational study, Chen et al. found that ceftazidime-avibactam had a significantly lower 30 day mortality rate when compared to polymyxin B (13.7% vs. 47.1%, respectively, *p* = 0.001) [136]. In two identical phase 3, multicentre trials of patients suffering cUTI (of which *E. coli* was responsible for 74.3%, and 24 were potentially carbapenem resistant) Wagenlehner et al. found cure in 77.4% patients treated with ceftazidime-avibactam vs. 71.0% doripenem patients, demonstrating non-inferiority of ceftazidime-avibactam [95]. Additionally, an international phase 3 trial of patients with cUTI and complicated intra-abdominal infections caused by ceftazidime-resistant *Enterobacterales* and *Pseudomonas aeruginosa* found that at test of cure, 91% (n = 140) of patients given ceftazidime-avibactam and 91% (n = 135) of patients given BAT had been cured [137]. Of these patients *E. coli* was responsible for 40–50% of infections [137]. Ceftazidime-avibactam was non-inferior to carbapenems, and a promising alternative.

## 4. Emerging Antibiotics

The development of new antibiotics is vital to the continued treatment of MDR UPEC infections. A variety of new antibiotics are in different stages of development, from undergoing phase 1 trials to completion of phase 3 trials [138]. While some antibiotics in development are only active against select few β-lactamases, others such as the combination of taniborbactam and cefepime have shown promising results across a wide variety of β-lactamases (Table 2).

### 4.1. Sulopenem

Sulopenem is a broad-spectrum synthetic penem which can be administered both orally and intravenously [9,138]. As a β-lactam, sulopenem works as a cell wall inhibitor against ESBL-producing cephalosporin resistant *Enterobacterales*, but it is not stable against carbapenemase [138]. Sulopenem has been shown to work potently against MDR *E. coli* in vitro [140]. Three phase 3 clinical trials spanning seven countries were completed between 2018–2020, however, the results were somewhat disappointing. The first study was an all-oral therapy study where sulopenem demonstrated inferiority to ciprofloxacin, and in the second and third studies IV sulopenem followed by orally administered sulopenem etzadroxil did not meet the non-inferiority margin, when compared to ertapenem [141,142,143].

### 4.2. Tebipenem

Tebipenem piyoxil hydrobromide (TBPM-PI-HBr) is a prodrug of the carbapenem tebipenem, promoted as a treatment for complicated UTI caused by MDR *Enterobacterales*, which have shown to be resistant to nitrofurantoin, sulfamethoxazole/trimethoprim, fluoroquinolones, and oral cephalosporins [141,144]. In a global, double-blind phase 3 trial (ADAPT-PO), 1372 patients received either TBPM-PI-HBr or ertapenem. Patients receiving TBPM-PI-HBr had an overall response rate of 58.8% compared to 61.6% response rate in patients receiving ertapenem. As TBPM-PI-HBr met non-inferiority requirements compared to IV ertapenem, it is a potential first or second line therapy for community acquired UTI [145].

### 4.3. Taniborbactam and Cefepime

Taniborbactam is a boronate-based BLI with inhibitory activity against ESBL, CTX-M, KPC-2 and 3, MBLs (especially NDM), and OXA-48. Cefepime is a fourth-generation cephalosporin. A global, randomized, double-blind, active-controlled non-inferiority phase 3 study was completed in late 2021. Preliminary findings showed that cefepime-taniborbactam had a clinical success in 70% of patients compared to just 58% of patients receiving meropenem. However, the complete results are yet to be released [146].

### 4.4. Enmetazobactam and Cefepime

Enmetazobactam is a penicillanic acid sulfone ESBL inhibitor, combined with cefepime to constitute an IV BLBLI combination, which presents an empiric, carbapenem sparing option for treatment of cUTI. Belley et al. conducted a phase 3 interventional, explanatory, double-blinded randomised non-inferiority clinical trial involving 1034 randomised patients over 19 countries, which ran from 2018–2021, and compared the efficacy and safety of enmetazobactam and cefepime with tazobactam and piperacillin [147]. Cefepime-enmetazobactam demonstrated a 79.1% efficacy at time of cure compared to 58.9% in piperacillin-tazobactam. Of the patients tested, 20.9% had ESBL producing uropathogens, 99.3% of which expressed a CTX-M type. Additionally, 3.5% co-expressed AmpC [147]. Cefepime-enmetazobactam presents another promising antibiotic.

### 4.5. Other Novel Antibiotics in Phase I or II Trials

Many other novel antibiotics are currently in phase 1 or 2 trials (Table 2). Novel diazabicyclooctane (DBO) β-lactamase inhibitors include, Zidebactam, effective against carbapenemases of Ambler classes A, B and D [148], nacubactam, shown to inhibit Class A, B and C and some Class D β-lactamases in vitro [149], ETX0282, an oral prodrug of ETX1317, with intrinsic antibacterial activity against *Enterobacterales*, active against ESBL, AmpC, OXA-48 and KPC producing strains, but ineffective against MBL producing strains [150], and ARX-1796, an oral prodrug of avibactam, a potent DBO active against KPC and OXA-48 but not MBL [150]. Additionally, other BLBLIs include Cefpodoxime-ETX0282, which may present a useful treatment for patients with ESBL producing pathogen causing UTI unresponsive to first-line agents [151], VNRX-7145 an oral boronate-based BLI active against Class A, C and D β-lactamases [152], and finally, xeruborbactam, which exhibits inhibition of metallo-β-lactamases of Classes A, B, C, and D in *Enterobacterales* [153].

## 5. Conclusions

Unsurprisingly, MDR bacteria represent a key public health concern worldwide. With UPEC responsible for 80–95% community acquired UTI cases and 27% sepsis cases, multi-drug resistance in UPEC remains a great concern [1,2,3,4,5]. Though some forms of multi-drug resistance in UPEC may be treatable, the appearance of carbapenem resistant, extended spectrum β-lactamase producing *E. coli* is a great concern. Treatment options for MDR UPEC are limited, though the development of novel BLBLIs presents promising future therapy options. Further research on the optimisation of existing treatment options as well as emerging novel agents in the development pipeline is constantly required to navigate treatment of these infections in a world of ever-increasing resistance.

## Figures and Tables

**Table 1:** Clinical studies evaluating the efficacy of antibiotics against numerous carbapenemase producing bacteria including *E. coli*.

Reference	Period	Design	Number of Patients	Objective	Treatment & Doses	Causative Agent/s	*E. coli* Specific Outcome	Outcome	ClinicalTrials.gov Identifier
[92]	July 2014 to July 2017	Phase 3 Multicentre Multinational Randomised Parallel Assignment Open-label Active controlled trial	77	To evaluate the efficacy and safety of meropenem–vaborbactam monotherapy against best available therapy for CRE.	Drug: Meropenem-vaborbactam 2 g/2 g dose via IV Drug: Best Available Therapy Antibiotic(s) chosen by Investigator	Confirmed or suspected carbapenem-resistant *K. pneumoniae*, *E. coli*, *Enterobacter cloacae* sp., *Proteus mirabilis*, *Serratia marcescens*	Not provided	Meropenem–vaborbactam cure rate of 65.6% versus 33.3% cure rate for best available therapy.	NCT02168946
[93]	2016 to 2019	Phase 3 Multicentre Randomised Parallel assignment Open-label Clinical trial	152	To evaluate the efficacy of cefiderocol for treatment of serious infections caused by carbapenem-resistant Gram-negative pathogens.	Drug: Cefiderocol 2 g intravenously over 3 h every 8 h for a period of 7 to 14 days, or 2 g every 6 h for participants with creatinine clearance >120 mL/min. Drug: Best Available Therapy Standard of care with either a polymyxin-based or non-polymyxin-based regimen as determined by the investigator and consisting of one to three marketed antibacterial agent(s).	Carbapenem resistant *Acinetobacter baumannii*, *K. pneumoniae*, *Pseudomonas aeruginosa*, *Stenotrophomonas maltophilia*, *Acinetobacter nosocomialis*, *E. cloacae*, *E. coli*	Mortality rate of cefiderocol was 17% vs. BAT 0%	Clinical cure achieved by 50% of cefiderocol patients and 53% of best available therapy patients suffering nosocomial pneumonia. Clinical cure achieved by 43% of cefiderocol patients and 43% of best available therapy patients suffering bloodstream infections or sepsis.	NCT02714595
[94]	2013 to 2016	Phase 4 Randomised Parallel assignment Open label Superiority trial.	406	To determine whether the addition of meropenem to colistin is superior to colistin monotherapy in the treatment infections caused by multi-drug resistant bacteria.	Drug: Colistin IV loading dose of 9 mil IU units Maintenance dose 4.5 mil IU q12h, adjusted for renal function, for 10 days. Drug: Meropenem IV 2 g every 8 h, adjusted for renal function, for up to 10 days. Drug: Colistin Loading dose of 9 mil IU units Maintenance dose 4.5 mil IU every 12 h, adjusted for renal function, for 10 days.	Carbapenem resistant *A. baumannii*, *Enterobacterales* and *Pseudomonas*	Not provided	No significant difference between colistin monotherapy and combination therapy was observed (79%, 73%, respectively).	NCT01732250
[95]	October 2012 to August 2014	A Phase 3 Multicentre Randomised Parallel assignment Double blind-double dummy Clinical trial	598 and 435 Combined total of 1033	To evaluate the effects of Ceftazidime Avibactam versus Doripenem for the treatment of cUTI	Drug: Ceftazidime-Avibactam (CAZ-AVI) Ceftazidime 2000 mg Avibactam 500 mg Every 8 h in a volume of 100 mL at a constant rate over 120 min administered IV. Drug: Doripenem 500 mg of Doripenem every 8 h administered by intravenous (IV) infusion in a volume of 100 mL at a constant rate over 60 min	Carbapenem resistant *E. coli*, *K. pneumoniae*, *Proteus mirabilis*, *E. cloacae*, *P. aeruginosa* and ESBL-positive *Enterobacterales*	Mortality rate of infections with: All baseline *E. coli* pathogens treated with ceftazidime-avibactam was 78.4% vs. doripenem 71.9% Ceftazidime-avibactam nonsusceptible *E. coli* treated with ceftazidime-avibactam 61.1% vs. doripenem 54.1% Ceftazidime-avibactam susceptible *E. coli* treated with Mortality rate of infections with 81.1% vs. doripenem 73.7%	Combined symptomatic resolution/microbiological eradication at test of cure was observed in 71.2% of CAZ-AVI patients vs. 64.5% doripenem patients.	NCT01595438 NCT01599806

**Table 2:** Expected activity of novel β-lactams and β-lactam-β-lactamase combinations against common β-lactamases [138,139].

	β-Lactamases			
	ESBL	KPC	OXA	MBL
Sulopenem	+	-	-	-
Taniborbactam + cefepime	+	+	+	+
Enmetazobactam + cefepime	+	?	-	-
Zidebactam + cefepime	+	+	+	?
Nacubactam + meropenem	+	+	+	+
ETXO282 + cefpodoxime	+	+	+	-
VNRX-7145 + ceftibuten	+	+	+	-
ARX-1796	+	+	+	-
Xeruborbactam + QPX2014	+	+	+	+

+ = antibiotic active against β-lactamase; - = antibiotic is not active against β-lactamase; ? = unknown if antibiotic is active against β-lactamase.
